# Actuation Strategies for a Wearable Cable-Driven Exosuit Based on Synergies in Younger and Older Adults

**DOI:** 10.3390/s23010261

**Published:** 2022-12-27

**Authors:** Javier Bermejo-García, Daniel Rodríguez Jorge, Francisco Romero-Sánchez, Ashwin Jayakumar, Francisco J. Alonso-Sánchez

**Affiliations:** Departamento de Ingeniería Mecánica, Energética y de los Materiales, Escuela de Ingenierías Industriales, Universidad de Extremadura, 06006 Badajoz, Spain

**Keywords:** motor control, wearable cable-driven exosuit, principal component analysis, gait analysis

## Abstract

Older adults (aged 55 years and above) have greater difficulty carrying out activities of daily living than younger adults (aged 25–55 years). Although age-related changes in human gait kinetics are well documented in qualitative terms in the scientific literature, these differences may be quantified and analyzed using the analysis of motor control strategies through kinetic synergies. The gaits of two groups of people (older and younger adults), each with ten members, were analyzed on a treadmill at a constant controlled speed and their gait kinetics were recorded. The decomposition of the kinetics into synergies was applied to the joint torques at the hip, knee, and ankle joints. Principal components determined the similarity of the kinetic torques in the three joints analyzed and the effect of the walking speed on the coordination pattern. A total of three principal components were required to describe enough information with minimal loss. The results suggest that the older group showed a change in coordination strategy compared to that of the younger group. The main changes were related to the ankle and hip torques, both showing significant differences (*p*-value <0.05) between the two groups. The findings suggest that the differences between the gait patterns of the two groups were closely related to a reduction in ankle torque and an increase in hip torque. This change in gait pattern may affect the rehabilitation strategy used when designing general-purpose rehabilitation devices or rehabilitation/training programs for the elderly.

## 1. Introduction

During aging, the capacity of older subjects to perform different actions, such as walking, stair climbing, or carrying out everyday activities, decreases [1,2,3]. One of the main causes is the loss of muscle mass associated with sarcopenia. This syndrome is characterized by a generalized and progressive loss of skeletal muscle mass that is often aggravated by physical inactivity and decreased mobility, leading to a slowing of the gait and a reduced ability to perform endurance exercises [4]. Sarcopenia represents a deterioration in health status and functional compromise due to movement disorders, which increase the risk of falls and fractures, impairment of the ability to perform activities of daily living (ADLs), loss of independence, and likelihood of death, leading to increased use of health services and thus increased socio-health expenditure [5]. This muscular weakness may lead to falls, most of which occur during the body-weight transfer phase from one leg to the other [6]. It is important to consider these changes in this group because balance and coordination start to decline at the age of 55, showing the need to understand how dynamic stability is affected [7,8,9].

The main changes in walking mechanics are manifested by a decrease in the ankle and hip joint torques [1,10,11]. This reduced ability to generate joint torques has been described as a limiting factor in walking speed and a modifier of walking patterns [12,13]. In addition, reduced motor ability has been described as a factor in musculoskeletal disorders and injuries [14]. Hence, understanding how the Central Nervous System (CNS) is organized to coordinate movement during walking is essential to determining its evolution with age-related changes in walking mechanics [3].

The CNS controls the human musculoskeletal system, which comprises more than 700 muscles and over 300 degrees of freedom (DOF) [15], giving us the capability to carry out complex movements. Due to high dimensionality, the motor control system must resolve a redundancy in order to provide coordinated and controlled movement with multiple DOFs [16]. This redundancy allows the adaptation to movements in constantly changing environments [17]. Variations in the number of DOFs used in the repetitions of a task are controlled by limiting the variability in their execution to a subspace of the total number available [18]. This suggests that during the execution of a specific task, patterns of coordinated behavior emerge to satisfy the objectives of that task, which are always subject to not only different environmental conditions but also physiological limitations such as age-related changes. In this context, invariant patterns identified as motor synergies are typical among all humans–patterns that define, for example, human gait [19]. The term synergy must be understood as a simplified organization of the control mechanism between limb segments [20,21]. The use of synergies allows human movement to be reconstructed using just a small amount of information registered during motion. The identification of these synergies provides objective information about how motor control strategies handle the execution of movement under different constraints. Some previous studies have ascertained that human gait can be explained by muscular synergies by analyzing the electromyography signal during walking [22,23,24]. Studies based on multi-segmental kinematic signals have also been carried out on human gait [22] and in varying locomotion tasks [25]. However, examining such kinetic variables as the force or net joint torque would provide a fuller comprehension of how the different motor behaviors develop under distinct constraints [26].

In order to identify dynamic synergies and how the neuromuscular system controls the execution of movements, principal component analysis has been used. Principal component analysis (PCA) has proven to be a powerful tool to reduce the dimensionality of data [27]. This multivariate, non-parametric statistical technique summarizes the most important information in the data through a limited number of components that explain the greatest amount of variance [21,27]. Several studies have compared various matrix factorization methods of reducing dimensionality and concluded that PCA outperforms other tools such as non-negative matrix factorization (NNMF) or independent component analysis (ICA) [28,29]. PCA has been applied for dimensionality reduction in research on walking [22,30] and postural balance [24,31]. The objective of most of these studies was to provide more information about the movement control mechanism [21].

**Figure 1:**
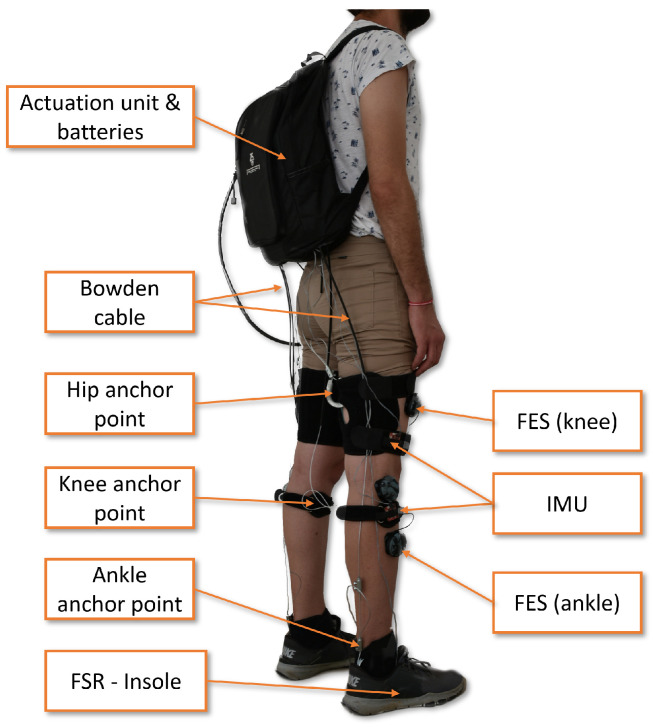
Exoskeleton developed for the project PID2019-107491RB-I00 (Spanish Agency of Research—MCIN/AEI/10.13039/501100011033). Sensors and actuators.

The present study aims to identify kinetic synergies to understand how motor control strategies vary under the constraints imposed by age-related changes. PCA is applied to provide a reduced dataset showing how movement develops under the aforementioned changes. A comparison of the gait patterns of younger and older adults at different speeds can provide a better understanding of how the motor control mechanism adapts in order to carry out a movement. We apply PCA to the net joint torque dataset to identify the most important characteristics needed to provide quantitative information about the mechanics of the age-related adaptations of the neuromuscular system. In particular, the aim is to characterize both gait patterns and identify the joint torques that are the most relevant in the execution of human gait. This fact is of great relevance when designing active exoskeletons or the more recent, lighter, and more wearable devices known as exosuits. The latter use wearable elements such as Bowden cables, straps, or pneumatic actuators to provide a pulling force able to move the limbs by following a control law (see [32] for further details on the design, [33] for sensors and [34] for current challenges). The work proposed here may provide insights into the design of such devices. In our previous works [35,36], we postulated and demonstrated that there were significant differences between kinematic and kinetic synergies in the sagittal plane that may affect the design of wearable exoskeletons, but they only affected the design of the transmission system. The exosuit design proposed in Figure 1 uses only one motor to assist both legs by using a pulley-based transmission system obtained through synergies (see [35] for further details). As the actuation was constrained in the sagittal plane, the question arises of whether it is necessary to include actuation in the coronal plane and whether this actuation is necessary for all those in the sample population or whether it can be simplified for some groups. The following sections describe the process for analyzing the gaits of the subject population that may benefit from this kind of device based on the use of synergies to reduce the number of actuators and, therefore, improve their wearability.

## 2. Materials and Methods

### 2.1. Participants

A dataset of 42 subjects reported by [37] was used in this study. Twenty healthy participants were chosen from that database and were assigned to a younger group (aged 18–40 years old) and an older group (at least 55 years old). As stated by Hafer et al. [7], changes in neuromuscular control that may affect segment coordination were identified in subjects who were 55 or older. These subsets were established to compare two groups of the same size once uncompleted trials were rejected. Table 1 summarizes the groups’, anthropometric data and gait speed. No significant statistical differences were found between the heights and weights of the subjects that may have affected this work. The subjects in the database had no record of lower limb musculoskeletal injuries or neurological disorders in the 6 months prior to the experiments that might have affected or modified their gait patterns at the time of the study.

Movement data were acquired using a motion capture system consisting of 12 cameras, 26 reflective markers [38], and an instrumented treadmill. The obtained raw marker trajectories and force platform data were processed using a low-pass Butterworth filter with a cutoff frequency of 10 Hz. For further details on the methods used and the ethical approval, see [37,39]. From this database, net joint torque data were selected and normalized to the gait cycle from 0% to 100%, starting from one heel contact to the next heel contact of the same foot. The data used were only for walking speeds of 55%, 100%, and 130%, as only ten out of twelve subjects in the older group in the database were able to complete all the trials.

### 2.2. Synergy Extraction

The PCA algorithm was used to identify kinetic synergies. It is a method for transforming correlated variables into a subset of uncorrelated variables that better exposes the various relationships between the original data elements. PCA also identifies and ranks the dimensions for which the points of the data show the greatest variations [27]. In this study, we performed PCA using the Singular Value Decomposition (SVD) algorithm. The input arrays contained all the net joint torque data for each subject (either in the younger adult group or older adult group). Each data series constituted a 540×101 matrix (rows: 10 participants × 3 trials × 18 torque measurements corresponding to 3D components at each of the 3 joints for the two legs; columns: 101 frames of the normalized gait cycle) $X_{i}\in {ℜ}^{540\times 101}$, where i=S,C,F represents each gait speed (S= slow, C= comfortable, F= fast). This input matrix was normalized by subtracting the mean and dividing it by the standard deviation of the data [22]. The *Scikit-Learn* Python package [40] was used to perform the standardization and PCA of the data series. The selection criterion was to include the number of PCs that explained more than 90% of the variance [41]. From the PCA, the principal component vectors (${PC}_{i}$) or the direction of the largest variation in the data matrix, the eigenvalues ($\lambda_{i}$) or the fraction of the total variance accounted for by each PC, and the scores [22,24] were obtained. The components determined the contribution of the original variable to a specific synergy. The loadings were expressed as absolute values.

To determine the number of PCs, the percentage variance explained or the variance accounted for (VAF) was used:

(1) $$\%\mathrm{VAF}=\frac{\sum_{i=1}^{m}\lambda_{i}^{2}}{\sum_{i=1}^{n}\lambda_{i}^{2}}\cdot 100$$

where *m* is the number of PCs used for the reconstruction (in this case, 3) and *n* represents the number of all eigenvalues. This parameter reflects the amount of variance that each PC explains.

### 2.3. Coordination among Joint Moments

Although the PCA trend contains as much of the variance as possible from the original dataset, the hierarchical clustering can be summarized in a dendrogram, where the leaves are the variables and the algorithm successively pairs objects showing the highest similarities.

To assess the interaction between the different joint moments, the correlation coefficient between each pair of joint moments was used. This coefficient provides information regarding possible changes in the synergies during aging [42]. In that case, the absolute value of the correlation coefficient shows the level of coordination between each pair of joint moments [43]. Using this value as the similarity measurement, the coordination relationships among the variables were studied using the agglomerate hierarchical clustering method. First, the average of the absolute values of the correlation coefficient was calculated across all participants. Second, by applying the average linkage algorithm, the similarity between two clusters can be defined.

### 2.4. Statistical Analysis

For the statistical analysis of the dataset, the *SciPy* library was used [44]. The Shapiro–Wilk test was used to evaluate the normality of the data. To assess the differences between the loadings for each PC, the data were compared using a *t*-test for the normally distributed data and a Mann–Whitney U-test for the equivalent non-parametric test. For all the tests, a two-sided *p*-value of less than 0.05 was taken as significant [45].

## 3. Results

The PCA model was applied to reduce the data’s dimensionality. Only three PCs were required to surpass the threshold of 90% of the variance explained. The importance of each PC is represented by the amount of variance it explains. The joint loadings can be used to interpret the role of each PC.

### 3.1. Movement Synergies Reconstruction

**Figure 2:**
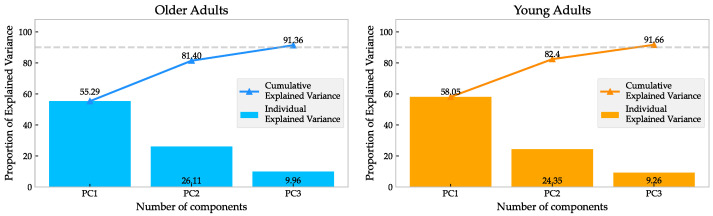
Percentage variance explained for each PC plotted individually in bars, and the cumulative variance explained with dots and lines for the older group (**left**) and the younger group (**right**). Only 3 PCs were necessary to describe the joint torques in adults.

Figure 2 shows the values of the VAF of each group. Only 3 PCs of 18 joint torques were required to reconstruct the dataset with at least a 90% VAF. In the case of the older group, the first PC accounted for 55.29% of the cumulative variance, the second PC accounted for 81.4%, and the third PC accounted for 91.36%. In the case of the younger group, the equivalent values were 58.05% for the first PC, 82.4% for the second PC, and 91.66% for the third PC.

In this sense, human walking, even at different speeds, has a common coordination pattern for the different elements that allow the said action. According to this description, the results suggest that lower limb joint moments could be reconstructed by a small number of PCs.

### 3.2. Loadings

**Figure 3:**
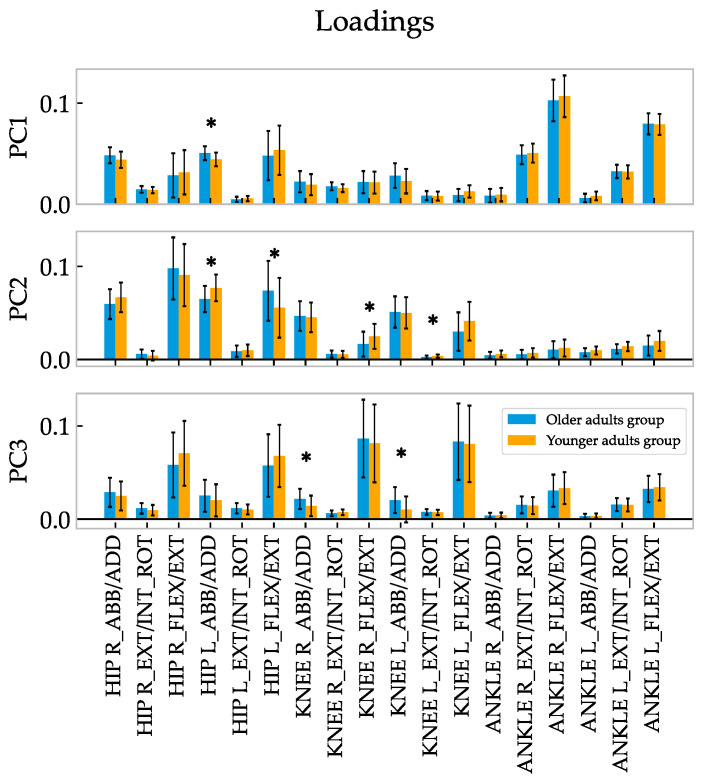
Loadings for each kinetic PC. R/L: right/left lower limbs; ABB/ADD, EXT/INT ROT, and FLEX/EXT represent the abduction/adduction moments, external/internal rotation moments, and flexion/extension moments, respectively. $\left({}^{*}p<0.05\right)$.

The PC loadings provide information on how the specific variables contribute to each PC. Analysis of the loadings showed a significant difference between the younger and older groups, as shown in Figure 3. The PC1 loading shows significant differences in the right hip abduction torques for both joints and the knee rotation for the right leg in the older group. In the case of the PC2 loading, differences were found between the loadings of the hip abduction moments for both legs and between the hip and knee flexion torques, showing lower values in the left (no dominant) hip moment for the older group. Furthermore, analysis of the PC2 values shows differences in the right knee flexion moment for the older group, where higher values were found. Similar results were observed in the case of the ankle abduction and rotation moments (see Figure 3). Analysis of the PC3 values reveals there were differences between the knee abduction moments for both lower limbs.

**Figure 4:**
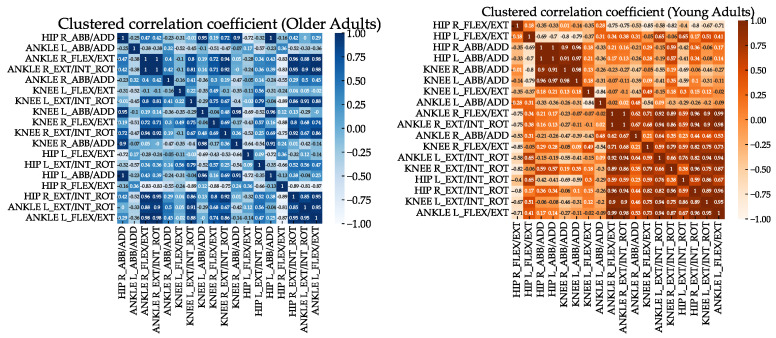
Clustered correlation heatmap for the older and younger groups.

**Figure 5:**
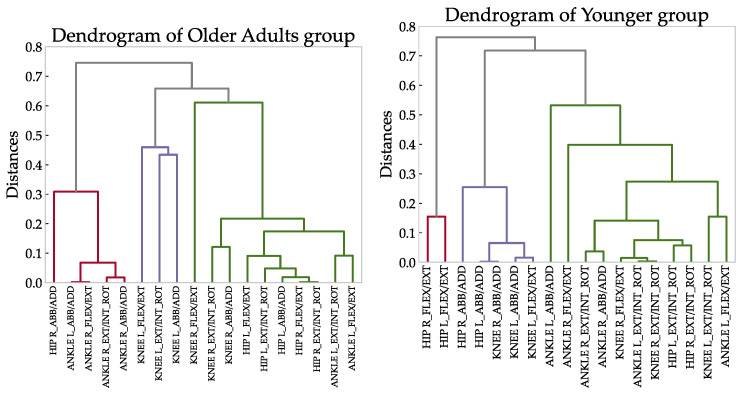
Dendrogram of the correlation relationship between the moment joints of the older group (**left**) and those of the younger group (**right**). The *Y*-axis shows the distances between the variables, where a lower node indicates a higher relationship between variables.

In accordance with the first synergy, the clustering analysis of the correlation coefficient (see Figure 4) demonstrated that there was a main coordinated subgroup consisting of ankle flexion/extension moments and ankle abduction/adduction moments, mainly of the dominant leg. Another subgroup, similar to the second and third synergies, was composed of the hip, knee moments, and ankle rotation moments. In the case of the younger group, some differences were observed. As shown in Figure 5, the ankle moments of the younger group are mainly grouped according to the first synergy. This group can be related to the other group composed of the knee flexion/extension moments and hip abduction/adduction moments according to the second synergy. By contrast, the hip flexion/extension moments are relatively independent of the other moments. Generally, humans adapt the moments during the gait cycle, indicating that the synergies are not just the result of matrix factorization (i.e., PCA) but represent some basic coordination characteristics within the lower limbs [42].

### 3.3. Application to the Design of Wearable Exosuits

Exosuits are a special type of exoskeleton in which actuation is provided using cables that pull the segments to which they are attached. In general, cables pass through trajectory points at any given instant during the gait as cable guides or follow a random trajectory if Bowden cables are used. As stated in [35], the desired flexion/extension torque at any specific instant obtained via inverse dynamics yields the cable forces required for the actuation as

(2) $$\tau_{j}=r_{AP,j}\cdot f_{j}$$

**Figure 6:**
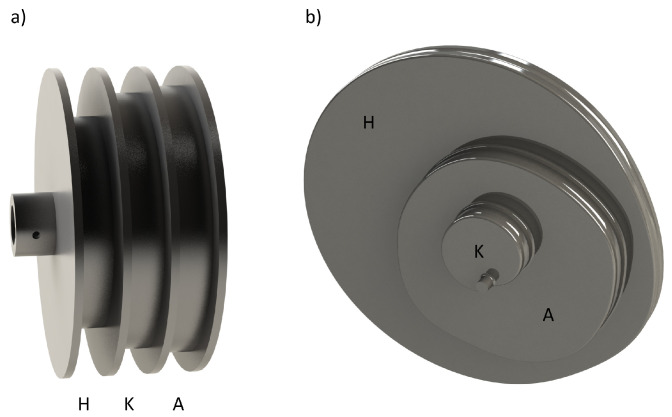
Transmission system for synergy-based actuation in exosuits. (**a**) Kinematic synergies. (**b**) Dynamic synergies. A: ankle; K: knee; H: hip.

In Equation (2), $\tau_{j}$ stands for the motor torque applied to joints *j* and *f* for the cable force, whereas $r_{AP,j}$ is a vector joining the joint *j* to the final anchor point. If PCA is conducted on kinematic data for the purpose of the exosuit to assist the gait to achieve a kinematically normative gait in the sagittal plane with the minimum number of actuation units, then the resulting transmission system leads to a set of pulleys able to reconstruct the gait within a range of VAF (Figure 6a). On the contrary, if PCA is conducted on kinetic data to provide the normative torques at each joint, then the transmission system results in a set of cams (Figure 6b).

**Figure 7:**
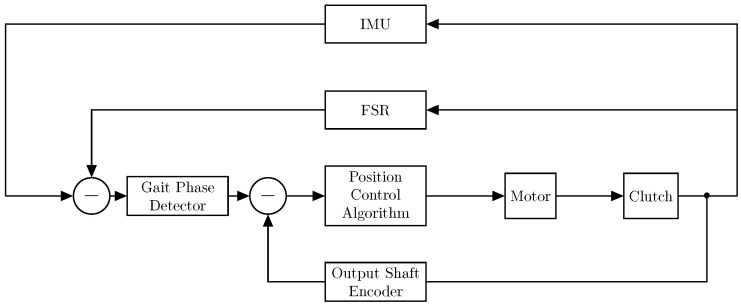
Control scheme for a synergy-based exosuit.

In this paper, out-of-plane torques were also included in the PCA study and thus may be considered to expand the synergy-based approach introduced in this document and further detailed in [36]. In such cases, there are two possible options for extending the actuation to out-of-plane motion and dynamics. First, by conducting a PCA study separately for the joint torques at each plane, which will yield a set of cams for each considered plane (in general, three sets for each selected PC and each leg). Although this may result in a more complex actuation system, it makes it viable to independently assess each actuation plane, increasing the number of selected PCs where needed. Second, by conducting a lumped PCA study on all planes, as conducted here, and developing a single set of cams, which will transmit forces to the joints via cables that are out of the sagittal plane. This latter option may yield a simpler actuation approach, although the variance accounted for might be lower for the first PC (given the larger amount of target variables), which means that the actuation might be less precise. Now, this must be considered when developing the control scheme and the pulleys/cams themselves. A more detailed analysis of a synergy-based design for wearable, assistive devices can be found in [36]. The general scheme to control the exosuit is depicted in Figure 7. The sensors used in the prototype for feedback were two IMUs on the leg, two force-sensing resistors (FSRs) on the heel, and an incremental encoder on the output shaft of the pulley. The gait phase detector used data from the inertial measurement units (IMUs) and FSRs to determine the current phase of the gait cycle in the sagittal plane and then sends the information to the position control algorithm. The position control algorithm additionally takes feedback from the output shaft encoder and sends the appropriate signal to move the motor and achieve the gait assistance required. The position controller also activates the clutches corresponding to the segment of the leg to be actuated.

An out-of-plane motion must include an extra IMU in the lumbar L4 position to account for tilting in the coronal plane. In this case, the cables act out of the sagittal plane, providing the actuation in both planes as stated before.

## 4. Discussion

This work aimed to identify kinetic synergies to understand how motor control strategies vary under the constraints imposed by age-related changes. As demonstrated above, joint torques were analyzed since these features are a direct reflection of the forces and torques that the soft tissues are exposed to, revealing the neuromuscular changes due to age. The PCA showed that the joint torques differed between younger and older adults at different gait speeds, highlighting the modifications of the walking strategy brought about by aging.

The PCA led to three control dimensions for each group. As mentioned, the first PC explained the greatest variance in the gait movement, followed by components 2 and 3 (see Table 2). Hence, for both groups, the first PC (PC1) was strongly related to the ankle flexion/extension net joint torque. The PC2 results for the two groups were similar, corresponding to a combination of the knee and hip joint torques in the frontal and sagittal planes, and PC3 showed common features associated with the knee flexion/extension joint torques. This interpretation would imply that PC1 explains the sagittal control dimension, whereas PC2 and PC3 combine the frontal and sagittal control dimensions. These results corroborate previous studies carried out on kinematic synergies (i.e., [22,46,47]). For example, ref. [46] proposed that walking patterns are composed of 3 PCs, where the first may correspond to stride length and the second to limb orientation.

Based on the present results, the main changes in the components were related to hip and ankle torques in the sagittal plane (see Figure 3). Similar results were reported in [48], where the effect of walking speed on the elderly was compared, concluding that the main changes were related to different shank-foot coordination with the aim of increasing postural stability [1]. The authors concluded that elderly people increase the force generated by their proximal leg extensor muscles. This is shown in Figure 3, where the main differences are related to hip torques in the frontal and sagittal planes, with the younger group’s loadings being higher than those of the older group in hip flexion/extension and ankle sagittal torques. These differences were also found in [9] where, at different gait speeds, hip extension and ankle plantar flexion reduced as the subject’s age increased. Similar results have been reported elsewhere in the literature [10]. This reduction when walking shows a change in walking strategy made by older people who tend to transmit forces through the hip joint by developing greater torques. These findings have been described as a limiting factor for elderly adults as they are obliged to adjust their walking pattern as the gait speed increases [11,12,13] or when climbing stairs [49].

The differences in the loadings of the second and third PCs were mainly related to hip flexion/extension and hip abduction/adduction. As shown in Figure 3, the older group had higher values related to hip flexion/extension. This redistribution of the role of the hip was due to compensating for the aforementioned differences in the ankle torque relative to the younger group in response to the increase in gait speed. These results agree with the descriptions in [13,50].

Lastly, it is worth considering that the present work, as well as that of [46], did not take into account the effects of the upper limbs. Although these effects were not contemplated in the objectives of this work, they should be included in future works, as lower limb control is strongly influenced by upper limb control, as evidenced in [47,51]. The results of this work can be used to rethink general-purpose rehabilitation therapies since compensatory differences in gait patterns that vary with age were observed. In the same way, although the design of gait assistance exoskeletons contemplates a specific design for each type of pathology associated with gait, generic gait assistance exoskeletons should include these differences in gait patterns to adapt the assistance to both target groups. This study could be extended in the future to analyze the gait compensatory patterns of healthy and injured/disabled subjects as the used database did not contemplate subjects with gait disabilities.

## 5. Conclusions

As we proved above, the walking pattern developed by the older group differed in several aspects from that of the younger group, especially in the execution of the hip and ankle joint torques. By applying PCA, the number of variables was reduced, thus simplifying the dimensionality of the problem. It was concluded that differences between the gait patterns of the two groups were closely related to a reduction in the ankle torque and an increase in the hip torque. These differences, especially in the production of the flexion/extension ankle torque, could affect the generation of an appropriate foot contact force and modify balance during walking, making the execution of the step less stable and reducing the normal gait speed.

## Figures and Tables

**Table 1:** Main characteristics of the study subjects.

		Young (N=10) (M±SD)	Older (N=10) (M±SD)
**Age** (years)		27.3±4.57	59.6±4.27
**Height** (cm)		171.9±8.55	161.65±9.2
**Weight** (kg)		68.35±9.5	66.36±11.14
**Speed** (m/s)	Slow	0.68±0.068	0.67±0.097
	Comfortable	1.23±0.12	1.26±0.2
	Fast	1.60±0.16	1.60±0.18

*M*: mean; *SD*: standard deviation; cm: centimeters; kg: kilograms; m/s: meters per second.

**Table 2:** Percentage of each individual (I) and cumulative (C) variance explained of each eigenvalue and qualitative interpretation of the first 3 PCs. Y/O: Younger and older groups.

*PC*	I-VAF(Y)	C-VAF(Y)	I-VAF(O)	C-VAF(O)	Characterization
1	58.05	58.05	55.29	55.29	Sagittal plane through the ankle joint
2	24.35	82.4	26.11	81.4	Frontal and sagittal plane through the hip and knee joints
3	9.26	91.66	9.96	91.36	Sagittal plane through the knee joint

## Data Availability

The data that support the findings of this study are openly available in [37] at https://pubmed.ncbi.nlm.nih.gov/29707431/ (accessed on 4 August 2022).
